# Optimized Deconvolutional Algorithm-based CT Perfusion Imaging in Diagnosis of Acute Cerebral Infarction

**DOI:** 10.1155/2022/8728468

**Published:** 2022-06-06

**Authors:** Xiaoxia Chen, Xiao Bai, Xin Shu, Xucheng He, Jinjing Zhao, Xiaodong Guo, Guisheng Wang

**Affiliations:** ^1^Department of Radiology, the Third Medical Centre, Chinese PLA General Hospital, Beijing 100039, China; ^2^Department of Geriatric, the Third Medical Centre, Chinese PLA General Hospital, Beijing 100039, China; ^3^Department of Dermatology, the Third Medical Centre, Chinese PLA General Hospital, Beijing 100039, China; ^4^Department of Neurology, PLA 305 Hospital, Beijing 100017, China; ^5^Department of Radiology, the Fifth Medical Centre, Chinese PLA General Hospital, Beijing 100039, China

## Abstract

To apply deconvolution algorithm in computer tomography (CT) perfusion imaging of acute cerebral infarction (ACI), a convolutional neural network (CNN) algorithm was optimized first. RIU-Net was applied to segment CT image, and then equipped with SE module to enhance the feature extraction ability. Next, the BM3D algorithm, Dn CNN, and Cascaded CNN were compared for denoising effects. 80 patients with ACI were recruited and grouped for a retrospective analysis. The control group utilized the ordinary method, and the observation group utilized the algorithm proposed. The optimized model was utilized to extract the feature information of the patient's CT images. The results showed that after the SE module pooling was added to the RIU-Net network, the utilization rate of the key features was raised. The specificity of patients in observation group was 98.7%, the accuracy was 93.7%, and the detected number was (1.6 ± 0.2). The specificity of patients in the control group was 93.2%, the accuracy was 87.6%, and the detected number was (1.3 ± 0.4). Obviously, the observation group was superior to the control group in all respects (*P* *<* 0.05). In conclusion, the optimized model demonstrates superb capabilities in image denoising and image segmentation. It can accurately extract the information to diagnose ACI, which is suggested clinically.

## 1. Introduction

Cerebral infarction is a cerebrovascular disease common in elderly people. It is always accompanied by chronic diseases, such as hypertension, hyperlipidemia, and diabetes. If not treated timely, it will seriously affect brain function. Epidemiological data show that acute cerebral infarction (ACI) accounts for about 50%–60% of all cerebrovascular diseases, whose mortality rate is 10%–15% [1]. Acute ischemic cerebral infarction is a common acute cerebrovascular disease, which mainly refers to the complete obstruction of the cerebral artery, the main trunk of the vertebrobasilar artery, and other branches, eventually resulting in the necrosis of the blood supply area of the brain tissue. Cerebral ischemia can cause irreversible damage to the function of brain tissue. The increase in infarct size will trigger a space-occupying effect. Acute cerebral infarction followed by hemorrhagic or hemorrhagic transformation is also one factor affecting its prognosis and treatment [2]. Related studies have found that the occurrence of ACI hemorrhage is associated with the integrity of the brain microvascular structure, that is, the blood-brain barrier. The increase of microvascular permeability in the ischemic area increases the amount of red blood cells leaking from the blood vessel [3]. Acute cerebral infarction has high fatality rate and disability rate [4]. Timely diagnosis is imperative for enhancing the prognosis [5].

CT examination can clearly show the size, location, and shape of ACI, and it is also economical, simple, and fast [6]. After CT examination, doctors can judge the treatment timing and treatment methods according to the size and number of the lesions [7]. Early CT detectors only performed perfusion scans at some slices, and failed to cover the whole brain, limiting their clinical applications and promotion. Dual-source computed tomography (DSCT) is a device under mature 64-slice CT, with a brilliant enhancement in time resolution, which is 83 ms, lower than 0.1s of cardiac imaging. CT scanning lifts the time resolution, and it has become a noninvasive diagnosis of cerebral infarction. Image feature extraction via different mathematical algorithms can position the lesion with a high running speed [8]. Dual-source CT is divided into plain scan, enhanced scan, and vascular examination. However, its definition was not high enough in traditional CT images. The inaccurate image segmentation also becomes a disadvantage in disease diagnosis. The application of intelligent algorithms in CT images could help display clear CT images and realize denoising. Many detection systems use deep networks to extract features for pixel classification. Convolutional neural networks (CNNs) are utilized in image segmentation, classification, and target positioning [9]. Reducing the size of the convolution kernel can faster the running speed of CNN [10]. The CNN was optimized in the study. RIU-Net was utilized to extract convolution features, equipped with the SE module to enhance feature extraction, and expected to provide novel approach for diagnosis of ACI.

## 2. Materials and Methods

### 2.1. Research Subjects

Eighty patients treated in the hospital from June 2018 to June 2020 were rolled into the control group (25 male + 20 female, (68.0 ± 3.2) years) and observation group (26 male + 14 female, (63.8 ± 3.4) years). This research had been approved of ethics committee of hospital, and the patients all signed the informed consent forms.

Inclusion criteria are as follows: (i) those diagnosed as ACI after examination; (ii) those having settled in the region for no less than 5 years; (iii) those admitted to the hospital within three days after the attack, and the examination interval between the two devices was within two hours; (iv) those having stroke for the first time; (v) those meeting the diagnostic criteria for ischemic stroke by the Fourth National Cerebrovascular Disease Academic Conference; and (vi) those having CT examinations during emergency visits.

Exclusion criteria are as follows: (i) those who cannot receive CT scan; (ii) mental illnesses such as vascular dementia and Lewy body dementia; (iii) incomplete clinical and imaging data; (iv) organ dysfunction; (v) patients with transient cerebral insufficiency and hypocalcemia convulsions; (vi) patients with severe organ complications; and (vii) patients with other types of cerebral ischemia.

### 2.2. Image Recognition

**Figure 1:**
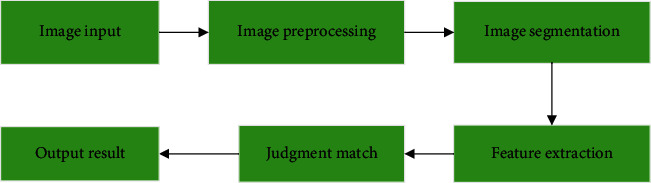
Traditional image recognition process.

Image recognition is an important branch of computer vision in which the computer analyzes the image and makes corresponding judgments. Humans get 90% of the information from the vision. With the advent of the computer era, deep learning for image recognition and judgment process also develops constantly, along with the development of the Internet.  Figure 1 shows the traditional image recognition process, mainly including the image input, preprocessing, feature extraction, and target recognition. Due to the complexity of image and the limitations of algorithms, the speed of image recognition is slow and the effect is not so satisfactory. The combination of artificial intelligence technology and image recognition has become a hot topic.

**Figure 2:**
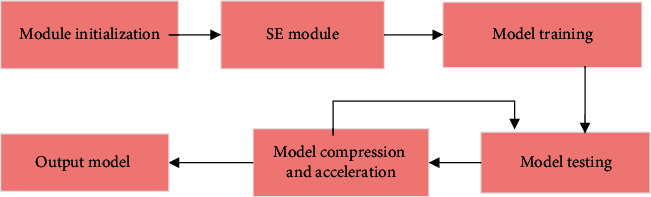
Schematic diagram of image recognition flowchart.

With the development of computer information technology, CNN arises. CNN demonstrates significant advantages in image and video identification using less pretreatment. It can learn and filter image features, and is widely utilized in natural language processing. In this study, the artificial neural network is incorporated in the existing image recognition process to optimize it. After image processing, feature extraction is performed. The artificial neural network model can reduce the complexity and redundancy, and SE module can enhance feature extraction. To include RIU network in SE can enhance the network accuracy.  Figure 2 presents the image recognition flowchart.

### 2.3. CNN

**Figure 3:**
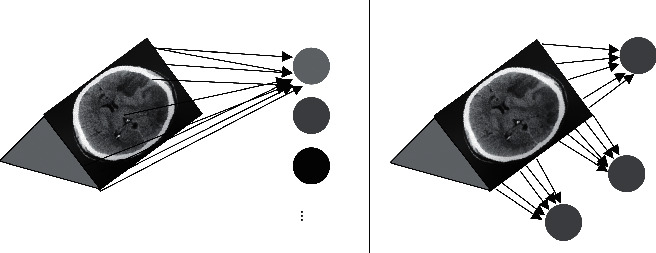
Schematic diagrams of full connection and convolutional form.

CNN is formed by fully connected networks. Each neuron is connected to another through input and output assigned weights, which are proportional to the number of neurons in each layer. Image is generally a three-dimensional matrix, including length, width, and the channel number. With multiple layers, CNN requires a huge amount of calculation. With the improvement of image pixels, the required weight matrix is also increasing. As the pixel of the input image grows to 1000 × 1000 and the number of neurons in the middle layer reaches 1M, 10^12^ pieces of data are needed to represent a simple two-layer fully connected network. In Figure 3, each neuron is connected to all the pixels in the image, and the matrix transformation is completed first. The pixel feature of a point is largely related to that of its surrounding pixels and less related to that of the point far away. The image itself has space information. After optimization, the weight coefficient of the CNN network is reduced, and the pixel is also increased.

After the convolutional layer and pooling layer of CNN are added to the network, the input layer is also different. Input layer input the three-dimensional image information as image input. If *H* is the height of an image, *W* is the width of it, and *C* is the number of input images, a color image with a size of 360 × 360 is represented as a column vector with a size of 360 × 360 × 3. CNN network with the three-dimensional input of 360 × 360 × 3 maintains the original spatial information of the image. The convolution layer obtains the eigenvalue matrix through the convolution operation. The input matrix is the current convolution layer's input. The convolution kernel refers to weight matrix of each layer in CNN, and the weight matrix is represented by the size of the weight from input matrix neuron to output matrix neuron.

**Figure 4:**
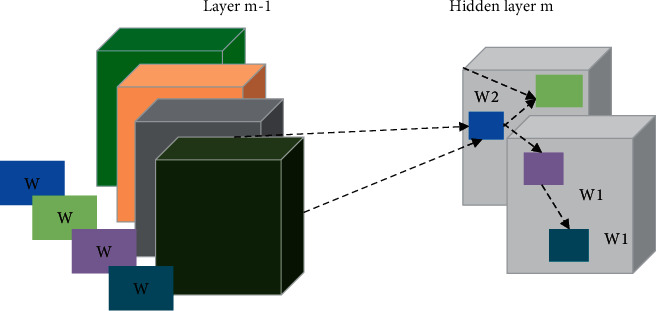
The calculation process of CNN.

The input matrix at layer *m* is the output matrix at layer *m*-1. The size of the image is H_in_ × W_in_ × 4.  Figure 4 shows the weight matrix. It is a 2 × 2 × 4×2 matrix, where 2 suggest 2 output channels, 4 indicates 4 input channels, 2 × 2 is the size of adjacent region corresponding to the input image. At W1, it is obtained by multiplying the 2 × 2 × 4 convolution kernel with the corresponding point of the matrix. The matrix features output by the CNN network are expressed as follows.

(1) $$\begin{array}{c} Ci,j,k=\sum_{m=\left(h/2\right)}^{m=\left(h/2\right)}\sum_{m=\left(h/2\right)}^{m=\left(h/2\right)}F\left(I_{i+m,j+n,k}w_{m,n,k}\right). \end{array}$$

After the convolution calculation is completed, the neuron completes the nonlinear operation in a complex way. F represents the activation function, and Sigmoid is generally utilized as the activation function. Different movements cause different neural units to be activated.

After the pooling layer inputs the feature map, the complexity of the calculation is reduced. The pooling of a convolutional kernel of *h* × *w* is expressed by (2). The parameters in the network are decreased, which in turn reduces the computation amount. Thus, it can maintain the invariance of the feature map for small shifts in the input.

(2) $$\begin{array}{c} \left(\frac{H}{h}\right)\times \left(\frac{W}{w}\right). \end{array}$$

Each output represents an expansion of the receptive field by a factor of *h* × *w*. The receptive field is expanded and then it enables the use of more surrounding information. The full connection layer is in line with the full connection of the neural network to ensure the consistency of output.

For the optimization of CNN network, the problems of fitting and underfitting need to be solved first so that the network can obtain the optimal performance in training. To make a set of weight parameters *Q* to approach the ideal weight *Q*^*∗*^, the generalization equation is as follows:

(3) $$\begin{array}{c} MSE\left(Q\right)=E\left({\left(Q^{*}-Q\right)}^{2}\right)=E\left({\left(Q^{*}-Q\right)}^{2}\right)+\text{VAR}\left(Q^{*}\right). \end{array}$$

**Figure 5:**
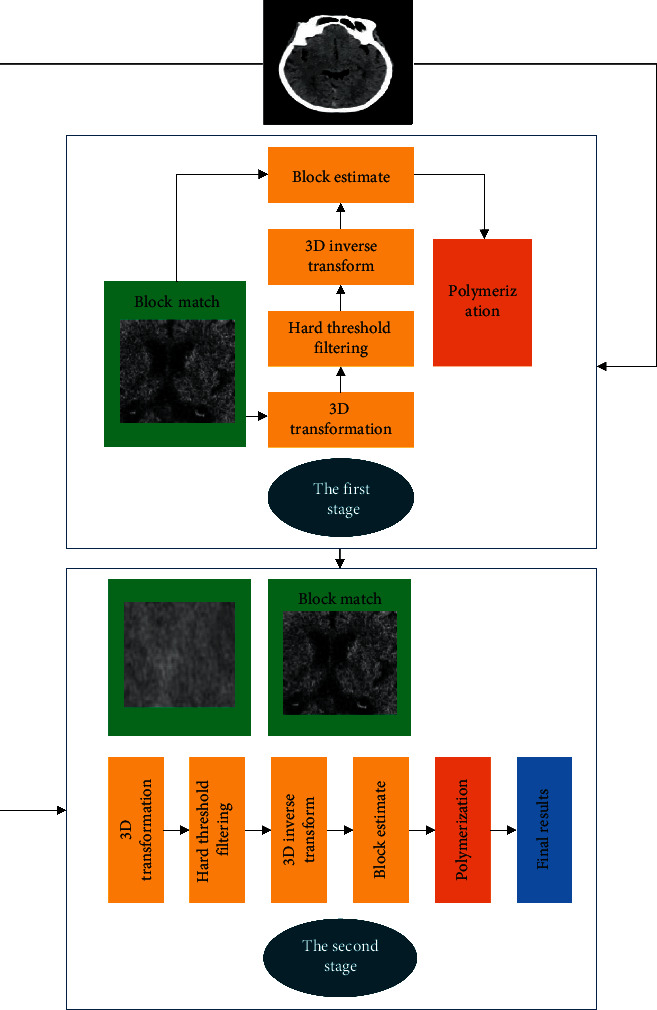
Schematic diagram of BM3D.

On the premise of ignoring noise, generalization error can be regarded as the combination of deviation and variance to measure the fitting ability of the network. A smaller deviation represents better fitting ability of the network to the data. The variance describes the overall disturbance of the network. A larger variance means that the network is more unstable and larger fluctuation in results (Figure 5).

### 2.4. Image denoising

**Figure 6:**
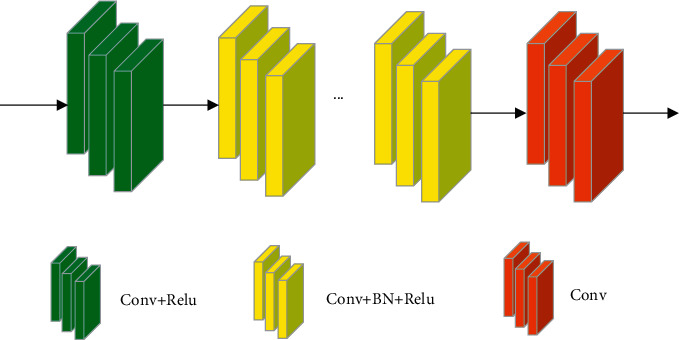
DnCNN structure.

There are many classic denoising methods, such as nonlocal denoising algorithms, full variational regularization, and block-matching and 3Dfitering (BM3D) algorithms. The overall operation of BM3D is relatively complicated. It mainly uses the similarity of image blocks to group them, and then carries out filtering operation on each image block to turn them to the two-dimensional form, and finally weighs and averages all similar images. The process is shown in Figure 6. It includes two stages. The first stage is to select reference blocks for the input image, and arrange all similar image blocks to form a three-dimensional matrix. Next, filtering is performed for two-dimensional transformation. Finally, the fused image blocks are put back in the original position. In the second stage, the original image and the result image are input. The three-dimensional matrix obtained is the same as that of the first stage.

### 2.5. Denoising Model

Denoising problems based on convolutional networks include IRCNN, FFDNet, RED-CNN, DnCNN, etc.

The denoising model expresses the low-dose CT image as follows:

(4) $$\begin{array}{c} \chi =\varphi \left(\text{y}\right). \end{array}$$

*χ*∈W^m×n^ represents a low-dose noise image, y ∈ W^m×n^ represents a normal image, and *φ*W^m×n^ is a mapping from a normal image to a noise image.

CNN-based image denoising is to find a function *f*, expressed as follows:

(5) $$\begin{array}{c} F=\arg\;\min{\left\|\text{F}\left(x\right)-y\right\|}_{2}^{2}. \end{array}$$

*F* is the approximate value of *φ*^−1^, and it represents the CNN. With more than two layers and sufficient parameters, the CNN can fit any functions. Theoretically, *F* = *φ*^−1^ exists.

### 2.6. DnCNN and Cascaded Structure

The structure of DnCNN is shown in Figure 6. DnCNN was originally utilized to process Gaussian noise. The network does not include any sampling process, and performs denoising directly on the original image to ensure the denoising effects.

**Figure 7:**
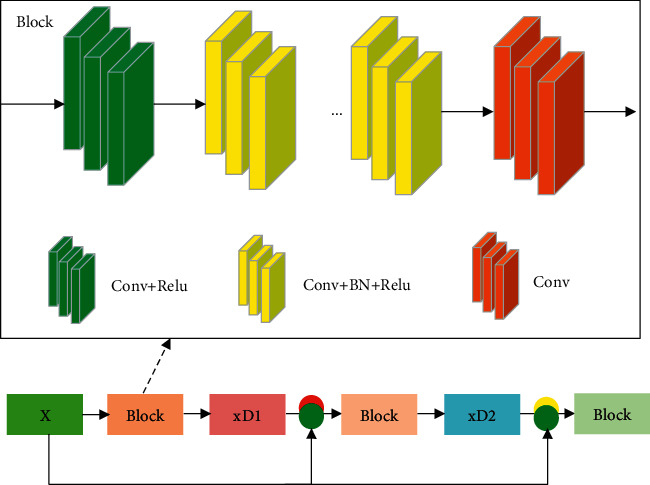
The Cascaded CNN structure.

In Figure 7, the DnCNN structure is optimized to a multilevel structure, namely, the Cascaded CNN structure. The denoising result of the previous layer and the original image are utilized as the input of the next layer. The artifact can be further processed in the next layer, and the next layer uses the original input information, which can well avoid the loss of information. A deeper number of BLOCKs can produce better performance.

### 2.7. Residual Neural Network Structure

If *X* is input in CNN, the arbitrary mapping of the network is expressed as *M(X)*. The ordinary convolutional network uses stacking learning *M(X)*, while the residual neural network (RNN) no longer directly maps *M(X)*, and the ideal mapping equation is as follows:

(6) $$\begin{array}{c} F\left(X\right)=M\left(X\right)-X. \end{array}$$

After the complex ideal mapping *M(x)* is converted into residual *F(x)* for learning, the learning objective between multiconvolutional layers is changed. When multilayer convolution operations are performed to approximate residuals, the network learns to output a residual function, *F*(*X*)=*M*(*X*) − *X*. If *F(x)* = 0, the optimization objective function approximates an identity mapping, rather than 0. To find the identity map is easier than to reproduce a mapping function. It suggests that compared to common network structure, RNN can learn information from more and deeper layers.

In feature extraction, the equations of information processing and image texture are shown as below.

Mean (i-mean) is defined as follows:

(7) $$\begin{array}{c} \bar{\text{x}}=\frac{1}{n}\sum_{i=1}^{n}x_{i}. \end{array}$$

Skewness (i-skewness) is calculated as follows:

(8) $$\begin{array}{c} D=\frac{\left(1/n\right)\sum_{i=1}^{n}{\left(x_{i}-\bar{x}\right)}^{2}}{{\left(\sqrt{\left(1/n\right)\sum_{i=1}^{n}{\left(x_{i}-\bar{x}\right)}^{2}}\right)}^{3}}. \end{array}$$

I-kurtosis is calculated as follows:

(9) $$\begin{array}{c} K=\frac{\left(1/n\right)\sum_{i=1}^{n}\left(x_{i}-\bar{x}\right)4}{{\left(\left(1/n\right)\sum_{i=1}^{n}{\left(x_{i}-\bar{x}\right)}^{2}\right)}^{2}}-3. \end{array}$$

**Figure 8:**
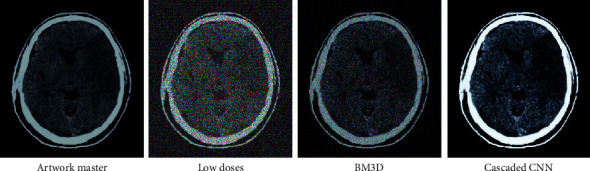
Comparison of CT images of the brain.

The loss function is optimized as below, where *F* is the actual data, and *E* represents the output predicted by the model. |E∩F| is an intersection of two sets. |E|, |F|, |E ∩ F| refer to the number size. *D1* is then calculated, as shown in Figure 8. The equations are as follows:

(10) $$\begin{array}{c} \text{DIce}=\frac{2\left|E\cap F\right|}{\left|E\right|+\left|F\right|},\\ =\frac{2\sum_{i}^{N}e_{i}f_{i}}{\sum_{i}^{N}{\text{e}}_{i}^{2}+\sum_{i}^{N}f_{i}^{2}},\\ {\text{DIce}}_{\text{distance}}=1-\frac{2\left|E\cap F\right|}{\left|E\right|+\left|F\right|},\\ =\frac{2\sum_{i}^{N}e_{i}f_{i}}{\sum_{i}^{N}{\text{e}}_{i}^{2}+\sum_{i}^{N}f_{i}^{2}}. \end{array}$$

The Dice model focuses on |E∩F|. The enhanced model avoids oversegmentation. Equation (11) shows the undersegmentation, and equation (12) is the oversegmented predicted outcome.

(11) $$\begin{array}{c} \text{New DIce}=\frac{2\left|E\cap F\right|-\left|\neg E\cap F\right|-\left|E\cap \neg F\right|}{\left|E\right|+\left|F\right|}, \end{array}$$

(12) $$\begin{array}{c} =\frac{2\sum_{i}^{N}e_{i}f_{i}-\sum_{i}^{N}\left(1-e_{i}\right)f_{i}-\sum_{i}^{N}e_{i}\left(1-f_{i}\right)}{\sum_{i}^{N}{\text{e}}_{i}^{2}+\sum_{i}^{N}f_{i}^{2}}. \end{array}$$

The change of the convolution structure is expressed in

(13) $$\begin{array}{c} \frac{H\times W\times D_{K}\times D_{K}\times M+H\times W\times M\times N}{H\times W\times D_{K}\times D_{K}\times M\times N}=\frac{1}{N}+\frac{1}{D_{K}\times D_{K}}. \end{array}$$

### 2.8. Scan Method and Scan Parameters

256-slice spiral CT was used. Patient was informed of the examination procedure and precautions in detail before scanning. Treatment plans for allergic reactions should be formulated in advance. The height and weight of the patient were accurately recorded before the examination, and a trocar was inserted into the anterior elbow vein. During the examination, the patient was instructed not to tilt the head, and not to speak or swallow to keep the body still, which can ensure good imaging results.

The head scanning requires a layer thickness of 5 mm, a tube voltage of 120 KV, and a tube current of 100 mA. The CT whole brain perfusion scan was performed under the reciprocating dynamic scan mode. The contrast agent was injected into the anterior elbow vein using a high-pressure syringe (double-barreled) at a flow rate of 4-5 mL/s, and 20 mL of normal saline was injected at 4-5 mL/s. The perfusion scan program began as the contrast agent was injected, with the tube voltage set to 80kv, and the tube current set to 125 mA. The matrix is 512 × 512, the scanning thickness is 5 mm, and the scanning is from the top of the brain to the side of the foot. The time is 45s, with about 20 cycles. After the scan, allergic reactions were observed in time, and the patient was asked to drink water to promote the discharge of the contrast agent.

The perfusion scan images were transferred to workstation, and processed by the deconvolution algorithm. The software automatically output the image of cerebral perfusion parameters, and then the abnormal perfusion area was divided by 3 physicians with over 5 years of experience. Any inconsistencies were solved through negotiation with a senior physician.

### 2.9. Image Segmentation

**Figure 9:**
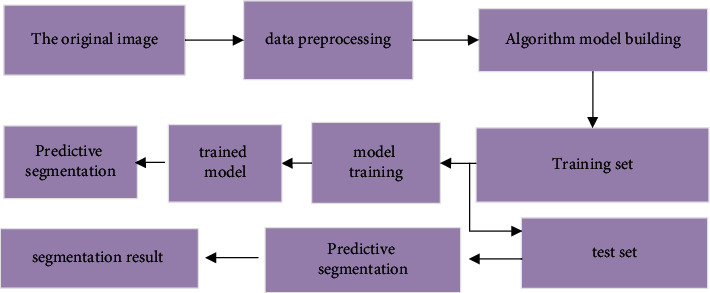
Flow chart of image segmentation.

Image segmentation can accurately diagnose diseases and locate lesions. Image segmentation and computer aided diagnosis can clearly identify the unclear area of the edge. A CT is a three-dimensional image of the human body. Region-based segmentation algorithm uses the gray uniformity of the same region to identify different regions of the image. Mapped to a standardized Montreal Neurological Society space, the image is divided into white matter, gray matter, and cerebrospinal fluid. Next, the target data is smoothed.  Figure 9 shows the flowchart of CT image segmentation by convolutional network. First, the original image is processed to segment the crane-less head, and then the image is standardized. Noise reduction and anticounterfeiting follow to obtain a smooth image, and finally the segmented image is obtained.

### 2.10. Statistical Methods

The data were processed by SPSS21.0. The measurement data (normal distribution) were the form of mean ± SD ($\bar{x}$ ± *s*), and relative frequency and frequency (%) were how nonconforming count data was indicated. The *T*-test was performed, *χ*^2^ test was performed for quality comparison, and *P* *<* 0.05 was the threshold for considerable difference.

## 3. Results

### 3.1. Image Comparison

Figure 8 showed the comparison of an original image and a low-dose image. It was found that the low-dose image had more particles than the original image, and the denoising effects were not good. BM3D and Cascaded CNN both exhibited obvious denoising effects, but BM3D was better. However, some areas were still not presented.

### 3.2. Performance Comparison on the Test Set

Table 1 listed the performance comparison between high-dose, low-dose, and BM3D denoising methods in a test set. BM3D parameter *φ* = 4. Other parameters are consistent with the simulation experiment.

**Figure 10:**
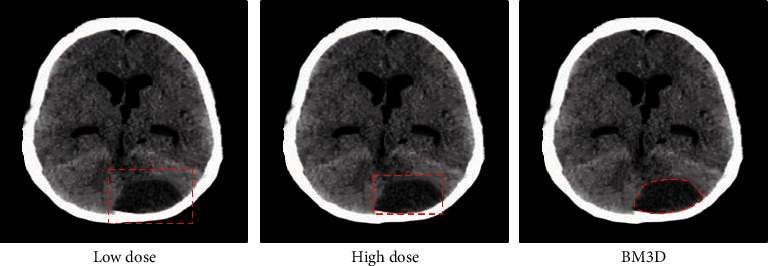
Slice effect diagram.

In Figure 10, the 115 slices of the CT image were analyzed. It was noted that the BM3D method was too smooth. The low-dose and high-dose white dots in the orange box were very light, and the white plots disappeared when using the BM3D method.

### 3.3. The Segmentation of Brain CT Images

**Figure 11:**
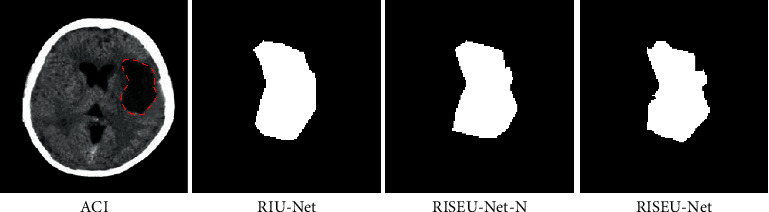
ACI CT images and segmentation results.

In Figure 11, the CT images of ACI, RISEU-Net network, RISEU-Net-N network, and RIU-Net network were used to segment the cerebral infarct area. The enhanced RISEU-Net network segmented CT image effectively.

### 3.4. Efficacy

The specificity, accuracy, and detected numbers were compared. In Table 2, the specificity, accuracy, and detected numbers in the observation group were superior to controls (*P* < 0.05).

## 4. Discussion

Both the incidence and mortality of cerebral infarction are on the rise [11]. The most common cerebral infarction is atherosclerosis, and the corresponding in situ thrombus leads to insufficient blood supply to brain tissue, resulting in neurological dysfunction. The brain is intolerant to ischemia and hypoxia. The blood supply interruption for 5 minutes will cause irreparable injury [12]. Imaging is an important part of the clinical diagnosis of ACI, and rapid and accurate diagnosis is a prerequisite for timely treatment [13]. It is reported that plain CT scan takes 22 hours to present the results of ACI [14, 15]. CT perfusion imaging is a functional imaging. Studies have found that abnormalities can be displayed after 30 minutes of cerebral ischemia. Some researchers use perfusion parameters to determine the severity of ACI, and use different algorithms to detect the efficacy. Different algorithms use different perfusion parameters, and there is a good correlation between the blood flow and blood volume of the diseased tissue measured by the deconvolution algorithm and the maximum slope method.

Deep learning is widely utilized. The evacuation design of buildings was simulated using a deep learning-based model, and the accuracy of the CNN model was verified by introducing auxiliary image prediction training and tracking sequence prediction training algorithm After image classification, the lesion's location is displayed clearly, and the features are extracted accurately [16, 17]. Shelhamer et al. (2017) utilized natural images to segment semantics [18]. Pereira et al. (2019) applied CNN in the segmentation of brain tumor images [19]. The specific locations in the convolutional layer were merged to create a new image [20, 21]. The optimized CNN algorithm was utilized, and RIU-Net was applied to segment CT image. The features of images could be clearly shown. CT images of the patients in observation group were clearer than those in the control group with less noise. Some scholars have applied the CNN in feature extraction. Coordinates are set in the lesion area to form a preselected detection frame, and then, the suspicious part is classified, which makes up for the shortcomings of the traditional method, and can detect the location of the lesion accurately. The model was optimized by deep learning [22]. The optimized model was defined as the RIU-net network, and it was then equipped with SE module, to enhance the feature extraction image [20]. The BM3D algorithm was compared with DnCNN and Cascaded CNN, and it was concluded that the BM3D algorithm had better denoising effects. SE module pooling was added into the RIU-Net network. The utilization of CT image features was remarkably enhanced. The specificity of the observation group reached 93.7%, while that of the control group was 87.6%. The result indicated that the optimized network showed significant capacities in image denoising and segmentation and could offer accurate information for ACI patients. The optimized network used in the research showed excellent effects in image denoising.

## 5. Conclusion

A CNN algorithm was enhanced and adopted for CT image segmentation of patients with ACI. Next, the SE module was supplemented to RIU-Net to enhance the extraction ability. It was found that the model's accuracy was 96.7% for image segmentation. The BM3D algorithm had better denoising effects compared to DnCNN and optimized Cascaded CNN. The deep learning model demonstrated superb capabilities in CT image denoising and segmentation. However, some limitations should be noted. The sample size was insufficient, and the segmentation did not reach 100%. Later, abundant samples are necessary to verify the findings of the study. In conclusion, a theoretical basis is given for extraction of lesion features in CT images [23, 24].

## Figures and Tables

**Table 1:** Comparison of denoising performance.

Method	PSNR	RMSE	SSLM
BM3D	32.46	9.786	32.68
Low dose	29.14	14.00	0.826
High dose	33.01	9.052	0.948

**Table 2:** Efficacy of two groups.

	Sensitivity (%)	Number of lesions detected	Accuracy (%)
Control group	93.2	1.3 ± 0.4	87.6
Observation group	98.7^*∗*^	1.6 ± 0.2^*∗*^	93.7^*∗*^

^*∗*^The difference was statistically significant (*P* < 0.05).

## Data Availability

The data used to support the findings of this study are available from the corresponding author upon request.
